# International comparison of spending and utilization at the end of life for hip fracture patients

**DOI:** 10.1111/1475-6773.13734

**Published:** 2021-09-07

**Authors:** Carl Rudolf Blankart, Kees van Gool, Irene Papanicolas, Enrique Bernal‐Delgado, Nicholas Bowden, Francisco Estupiñán‐Romero, Robin Gauld, Hannah Knight, Olukorede Abiona, Kristen Riley, Andrew J. Schoenfeld, Kosta Shatrov, Walter P. Wodchis, Jose F. Figueroa, ICCONIC Collaboration

**Affiliations:** ^1^ KPM Center for Public Management University of Bern Bern Switzerland; ^2^ Swiss Institute of Translational and Entrepreneurial Medicine Bern Switzerland; ^3^ Hamburg Center for Health Economics Universität Hamburg Hamburg Germany; ^4^ Centre for Health Economics Research and Evaluation (CHERE) University of Technology Sydney Australia; ^5^ Department of Health Policy and Management Harvard T.H. Chan School of Public Health Boston Massachusetts USA; ^6^ Department of Health Policy London School of Economics London UK; ^7^ Institute for Health Sciences in Aragon (IACS) Zaragoza Aragon Spain; ^8^ Department of Women's and Children's Health University of Otago Dunedin New Zealand; ^9^ Otago Business School and Centre for Health Systems and Technology University of Otago Dunedin New Zealand; ^10^ The Health Foundation London UK; ^11^ Division of Orthopedic Surgery Brigham & Women's Hospital, Harvard Medical School Boston Massachusetts USA; ^12^ Institute of Health Policy Management & Evaluation University of Toronto Toronto Ontario Canada; ^13^ Institute for Better Health, Trillium Health Partners Mississauga Ontario Canada

**Keywords:** administrative data, age inequalities, end‐of‐life care, gender inequalities, health care spending, health care utilization, international comparison

## Abstract

**Objective:**

To identify and explore differences in spending and utilization of key health services at the end of life among hip fracture patients across seven developed countries.

**Data Sources:**

Individual‐level claims data from the inpatient and outpatient health care sectors compiled by the International Collaborative on Costs, Outcomes, and Needs in Care (ICCONIC).

**Study Design:**

We retrospectively analyzed utilization and spending from acute hospital care, emergency department, outpatient primary care and specialty physician visits, and outpatient drugs. Patterns of spending and utilization were compared in the last 30, 90, and 180 days across Australia, Canada, England, Germany, New Zealand, Spain, and the United States. We employed linear regression models to measure age‐ and sex‐specific effects within and across countries. In addition, we analyzed hospital‐centricity, that is, the days spent in hospital and site of death.

**Data Collection/Extraction Methods:**

We identified patients who sustained a hip fracture in 2016 and died within 12 months from date of admission.

**Principal Findings:**

Resource use, costs, and the proportion of deaths in hospital showed large variability being high in England and Spain, while low in New Zealand. Days in hospital significantly decreased with increasing age in Canada, Germany, Spain, and the United States. Hospital spending near date of death was significantly lower for women in Canada, Germany, and the United States. The age gradient and the sex effect were less pronounced in utilization and spending of emergency care, outpatient care, and drugs.

**Conclusions:**

Across seven countries, we find important variations in end‐of‐life care for patients who sustained a hip fracture, with some differences explained by sex and age. Our work sheds important insights that may help ongoing health policy discussions on equity, efficiency, and reimbursement in health care systems.

**What is known on this topic**

- Countries follow different approaches to providing end‐of‐life care.
- Health care spending and utilization increases near death.
- International studies of health systems mainly compare acute hospital care and therefore lack to account for different organization of end‐of‐life care.

**What this study adds**

- End‐of‐life care in the United States occurs more with specialists than in primary care, which is in fundamental contrast to other countries.
- In most countries, health care spending and utilization decreases with age and is lower for women.

## 1 INTRODUCTION

Health policy makers have a strong interest in developing a better understanding of how they can improve end‐of‐life care.^1^, ^2^, ^3^ Prior studies have found that in the months leading to death, the need and the costs of care increase substantially, and the bulk of these expenditures come from high‐acuity, high‐cost individuals, such as those with persistent chronic conditions.^4^, ^5^, ^6^ In addition, there are serious concerns that the quality of care at the end of life often falls short of expectations.^7^, ^8^, ^9^ As more people reach old age with chronic and disabling conditions, improving the quality and efficiency of care at the end of life will continue to grow as a priority policy issue.^10^ As such, end‐of‐life care has become an important dimension of how we measure health system performance.^11^

International comparisons on end‐of‐life care may yield important insights into how policy makers could improve the efficiency and the quality of care. Such research is vital in setting performance benchmarks and establishing best practice models from a system‐wide and policy perspective. However, to date, comparisons at the end of life across countries are quite limited, especially when it comes to comparing robust data across more than two countries and across different health care sectors.

In recent years, there have been significant advances in data infrastructure across many countries that enable international comparative research. This development is particularly true for patient‐level data, which is necessary to examine potential differences in end‐of‐life care. Much of the relevant data are routinely collected through administrative datasets, which are increasingly accessible for research and quality improvement purposes.^12^ For example, claims data from health care payers, such as health insurers or national health services, provide a solid starting point for international comparisons as shown in several international projects such as Health Basket^13^ and EuroDRG.^14^

In this study, as part of the International Collaborative on Costs, Outcomes, and Needs in Care (ICCONIC) project, which is a research collaborative across a set of high‐income countries, we sought to evaluate differences in treatment at the end of life among frail, older adults who sustained a hip fracture across seven countries as follows: Australia, Canada, England, Germany, New Zealand, Spain, and the United States. Specifically, we sought to examine differences in utilization and spending across key health care services, including hospital care, emergency care, primary care and outpatient specialty care, and pharmaceuticals. Using a framework analyzing hip fracture patients, our goal was to provide insight into how health systems can optimally address the needs of the frail decedents by effectively accounting for resource constraints.

## 2 SUMMARY OF PREVIOUS INTERNATIONAL STUDIES ON END‐OF‐LIFE CARE

There is an extensive literature on the health care costs associated with end‐of‐life care. Numerous studies have shown that health care costs increase manifold in the time leading up to death.^15^, ^16^, ^17^ Riley and Lubitz,^18^ for example, find that although decedents account for only 5% of the US Medicare population in any given year, expenditure on this group explains more than 25% of total Medicare expenditure. As has been shown, the bulk of these expenditures come from high‐need high‐cost individuals, such as those with persistent chronic conditions.^19^

The high costs at the end of life have led the policy makers to question if health systems are obtaining value for money in end‐of‐life care—particularly when considering that quality of care remains far from optimal.^20^, ^21^, ^22^, ^23^ At the same time, there is growing evidence that not all patients at the end of life face the same cost trajectory.^24^, ^25^, ^26^ These studies show that there is considerable heterogeneity in the pattern in health care use and associated costs at the of end of life among different patients. Some patients face very high and persistent costs over an extended period of time, and others face a sudden decline in health status and associated rise in health care costs over a very short period of time.^27^ As a result, there are widening calls to develop a greater understanding the drivers of end‐of‐life costs.

Another area of importance to health policy makers is awareness of gender and age disparities, including in end‐of‐life care. While literature found gender disparities in end‐of‐life spending and utilization, the direction of the disparity is not always consistent.^28^, ^29^, ^30^ Some studies show that women receive less aggressive treatment than men receive when it comes to cancer or care in intensive care units. One potential reason is that women are more likely to have a do‐not‐resuscitate order than men.^31^, ^32^, ^33^ In addition, other work has found that end‐of‐life spending at least in the United States declines with age, indicating declining treatment intensity.^28^ Thus, while there is some evidence in the area of cancer care, the evidence for frail elders with a hip fracture, while similarly important, is less established. Further, cultural factors can influence utilization of health services. In some countries, there is variation in the proportion of older adults who live alone versus live with other family members. In the United States, evidence suggests that older adults are more likely to live alone than other countries and, therefore, may have a limited support system to care for themselves safely at home (and thus end up in a skilled nursing facility).^34^ We therefore aim to expand on this literature and, importantly, show some consistency in patterns across countries by these important demographics.

Health policy makers who shape the health system have a strong interest in understanding how different countries provide end‐of‐life care, including current work at the Organisation for Economic Co‐operation and Development (OECD). The international evidence on end‐of‐life care is limited—especially when it comes to comparing robust data across more than two countries. There are two notable exceptions. The first is French et al.^5^ who estimated the cost of care in the last 30 and 365 days of life across nine countries. This study found that mean cost in the last 12 months of life varied from US$ 50,000 in Germany to over US$80,000. Noting that the study was unable to control for the cause of death nor report on utilization. The second exception is Bekelman et al.^7^ who examined hospital and chemotherapy use and cost in seven countries in the last 180 days of life for patients who died with cancer. The study found that mean per capita hospital expenditures were highest in Canada (US$ 21,840) and lowest in England (US$ 9342). Whilst Bekelman et al.^7^ focused on a relatively homogenous patient group, the study was restricted to estimating costs in a sub‐set of the health care sector—albeit very important sector.

This project builds on previous work and makes a considerable contribution to developing a better understanding of internationally comparable end‐of‐life costs and health care use. To the best of our knowledge, this is the first study that has examined the end‐of‐life costs for frail elderly patients who have experienced a hip fracture in the last year of their life. This focus will provide new insights into end‐of‐life costs for a relatively homogeneous group of frail elderly patients who—due to their clinical condition—may be less likely to be cared for in a palliative care setting.

Frail elderly hip fracture patients are a vulnerable patient group with a high mortality rate following on from their hip fracture^35^ and likely to incur significant health care costs.^36^ Using standardized codes to identify a relatively frail and homogeneous group of patients, this article reports on a broad range of health care costs and utilization measures in seven countries and develops country‐specific estimates of these costs at various time points in the months leading up to death.

## 3 METHODS

### 3.1 Data sources

We used patient‐level data of seven out of the eleven countries participating in the ICCONIC collaborative, that is, all countries that could determine the exact date of death, to examine end‐of‐life utilization, and spending as follows: Australia (AU), Canada (CA), England (EN), Germany (DE), New Zealand (NZ), Spain (ES), and the United States (US). Extracted utilization and spending data of Australia, Canada, Germany, Spain, and the United States are based on individual‐level claims data from 2015 to 2017, while England and New Zealand used a longer identification period. For specific details of each country's dataset, please see Appendix 1 and Figueroa et al.^37^

### 3.2 Patient selection

We followed a two‐step approach to identify a comparable set of frail elders who received treatment for a hip fracture. Hip fracture has been commonly used as a reliable marker of frailty among older adults,^38^ and it accounts for the majority of fractures related to fragility globally.^39^ Hip fracture is also highly associated with physical and mental disability, high mortality, and increased costs, thus requiring considerable health care resources from different parts of the health system.^39^, ^40^, ^41^, ^42^ As hip fractures almost always require a hospital admission and usually require surgery, the majority will be recorded in hospital admissions data and can thus serve as a robust and reliable tracer condition to explore differences in resource use across health systems.^38^ We first identified a sample of comparable patients by examining all patients who received a primary diagnosis of hip fracture (S72.0‐2 according to the International Classification of Diseases version 10) in 2016 and obtained a total hip replacement, a partial hip replacement, or were treated with an osteosynthesis method such as screw, plate, pin osteosynthesis, or internal fixation (see Appendix 2). From this sample, we identified those who died within 365 days from the index hospitalization associated with the hip fracture.

### 3.3 Spending and utilization measures

**FIGURE 1:**
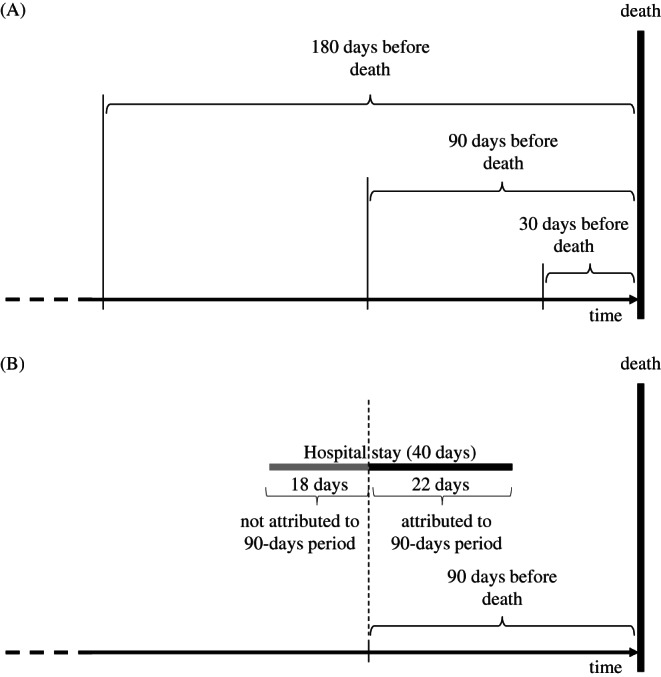
(A) Utilization and spending included in the analyses of 30, 90, and 180 days before death and (B) calculation of utilization and costs in case of accruals at the example of the 90‐day period

We followed a federated data extraction approach due to data protection reasons. Each country produced means of utilization and spending by sex and age (65–69, 70–74, 75–79, 80–84, 85–89, 90–94, and older than 95 years), from individual patient‐level data. These aggregated nonidentifiable data were then collected in a central database for the analysis. It is important to note that we used the perspective of the health care payer across all countries. In most countries, this is either directly by an insurance or sickness fund (Germany and the Netherlands) or directly from a national form of health insurance (United States with Medicare program, Canada, etc.). Therefore, our study does not capture full costs (as it does not account for the fixed costs of all structures within a health system). It only captures were actually paid for the services, which across all countries, already included the fixed costs of the system. In order to compare spending reliably, we first applied the OECD Actual Individual Consumption Purchasing Power Parities (AIC PPPs) to the expenditure data. AIC PPPs, rather than gross domestic product–based purchasing power parities, are currently used by the OECD as the most reliable economy‐wide conversion rates for health expenditure.^43^ Across each country, we applied 2017 AIC PPPs to all expenditures using the following exchange rates as follows: 1 AU$ ≙ US$ 0.69, 1 CA$ ≙ US$ 0.83, 1 GBP ≙ US$ 1.45, 1 EUR (Germany) ≙ US$ 1.33, 1 NZ$ ≙ US$ 0.69, 1 EUR (Spain) ≙ US$ 1.56, and US$ 1 ≙ US$ 1.^37^ Similar to Bekelman et al.,^7^ we calculated average utilization and average spending for acute hospital care, emergency department admissions, excluding observation stays, primary and specialist outpatient physician services, including the services of nurse practitioners in Australia, Canada, and the United States and outpatient drugs for the periods of 30, 90, and 180 days before death (see Figure 1A). Utilization and spending were allocated proportionally to the observation periods in case of accruals (see Figure 1B).

### 3.4 Data analysis

First, we described the number of decedents by country and sex. From each country's total sample of elderly patients who have experienced a hip fracture, we calculate the proportion of people who have died within 365 days of the fracture.^44^ We also calculated the proportion of decedents in hospital relative to those who are discharged after their hip fracture admission.

To analyze within‐ and between‐country variation, we estimate utilization and spending $y_{i}$ as a function of country‐fixed effects to determine baseline utilization and baseline spending, respectively. In addition, we include the interaction of country and sex, the interaction of country and age group, which we coded ascendingly from 1 to 7 for the 65–70 years to the older than 95 group, and the interaction of country and days before death into the model to disentangle potential country‐specific effects of sex, age, and time. We fitted the model with no intercept term and, therefore, the age group and country estimates refer to zero, that is, we needed no reference group. We define the following six dependent variables for utilization as follows: acute hospital admissions, days in hospital, emergency department visits, medical doctor specialist visits, primary care visits, and outpatient prescription drugs. In addition, we measure the following five dependent variables spending as follows: acute hospital stays, emergency department, specialists, and primary care visits, as well as for outpatient prescription drugs. We estimate for each dependent variable the following linear regression:

$$y_{i}=x_{i}\beta \;+\;x_{i}\;\mathrm{sex}\;\phi \;+\;x_{i}\;\mathrm{age}\;\gamma \;+\;x_{i}\;\text{time}\;\tau \;+\;\varepsilon$$

where $x_{i}$ denotes a vector of country‐fixed effects and ε is a normally distributed error term. We obtaine β indicating baseline utilization and spending, ϕ sex‐specific utilization and spending, γ an age gradient, and τ time‐dependent utilization and spending. We considered p<0.05 as statistically significant throughout the whole paper. All estimations were using the statistical program R 4.0.3 and the integrated lm function for linear regression.^45^

## 4 RESULTS

### 4.1 Descriptive statistics

A total of 16,482 decedents were observed across the seven countries. In relation to the main cohort,^37^ between 23.0% and 31.6% of all frail elders died within 365 days after a hip fracture across countries. Across all countries, more women (10,588) than men (5894) died after the event in absolute terms, but the relative mortality of women was lower than that of men. Mortality ranged for women between 20.3% and 28.2% and for men between 29.5% and 40%. Next, we observed distinct differences in hospital‐based mortality: while in Spain, 40% of patients died in hospital, in Australia, this occurred in only 6.7% of patients. Across all countries, men had a higher likelihood of dying in hospital (range 6.3%–49.7%) than women (range 7.1%–35.2%), while the variation across age groups was not pronounced (Table 1).

**TABLE 1:** Number and percentage of decedents by country, age group, and site of death

	Australia			Canada			England			Germany			New Zealand			Spain			United States		
	All	F	M	All	F	M	All	F	M	All	F	M	All	F	M	All	F	M	All	F	M
*Baseline population (as reported in ICCONIC project)*																					
All age groups	2511	1577	934	9872	6975	2897	2738	1943	795	13,998	10,628	3370	2940	2070	870	1849	1419	430	29,134	20,789	8345
AG 65–69	141	93	48	695	425	270	166	102	64	759	516	243	168	105	63	62	45	17	2099	1314	785
AG 70–74	197	127	70	931	603	328	239	158	81	1085	761	324	246	165	81	81	57	24	3024	2032	992
AG 75–79	298	191	107	1341	898	443	387	285	102	2455	1810	645	369	255	114	164	123	41	4180	2877	1303
AG 80–84	455	281	174	1946	1361	585	627	432	195	2902	2137	765	573	411	165	404	305	99	5711	4028	1683
AG 85–89	751	460	291	2498	1815	683	666	480	186	3385	2567	818	831	579	249	602	467	135	6840	4960	1880
AG 90–94	523	320	203	1832	1374	458	462	335	127	2578	2120	458	555	399	156	412	317	95	5221	3929	1292
AG 95+	146	105	41	629	499	130	191	151	40	834	717	117	198	156	42	124	105	19	2059	1649	410
*Number of decedents*																					
All age groups	640	324	316	2272	1416	856	865	547	318	3662	2505	1157	798	522	276	473	318	155	7772	4956	2816
AG 65–69	10	4	6	72	34	38	22	‐	‐	76	40	36	‐	‐	‐	11	8	3	312	168	144
AG 70–74	18	9	9	123	69	54	47	‐	‐	136	69	67	‐	‐	‐	9	7	2	454	264	190
AG 75–79	53	29	24	220	120	100	75	50	25	434	271	163	66	36	30	28	16	12	789	447	342
AG 80–84	97	48	49	408	236	172	171	99	72	647	411	236	135	84	51	71	43	28	1359	816	543
AG 85–89	208	102	106	592	363	229	237	146	91	988	642	346	255	171	84	155	103	52	2103	1323	780
AG 90–94	186	87	99	582	388	194	201	128	73	971	734	237	216	144	72	145	99	46	1847	1251	596
AG 95+	68	45	23	275	206	69	112	84	28	410	338	72	84	63	21	54	42	12	908	687	221
*Percentage of decedents*																					
All age groups	25.5%	20.5%	33.8%	23.0%	20.3%	29.5%	31.6%	28.2%	40.0%	26.2%	23.6%	34.3%	27.1%	25.2%	31.7%	25.6%	22.4%	36.0%	26.7%	23.8%	33.7%
AG 65–69	7.1%	4.3%	12.5%	10.4%	8.0%	14.1%	13.3%	‐	‐	10.0%	7.8%	14.8%	‐	‐	‐	17.7%	17.8%	17.6%	14.9%	12.8%	18.3%
AG 70–74	9.1%	7.1%	12.9%	13.2%	11.4%	16.5%	19.7%	‐	‐	12.5%	9.1%	20.7%	‐	‐	‐	11.1%	12.3%	8.3%	15.0%	13.0%	19.2%
AG 75–79	17.8%	15.2%	22.4%	16.4%	13.4%	22.6%	19.4%	17.5%	24.5%	17.7%	15.0%	25.3%	17.9%	‐	‐	17.1%	13.0%	29.3%	18.9%	15.5%	26.2%
AG 80–84	21.3%	17.1%	28.2%	21.0%	17.3%	29.4%	27.3%	22.9%	36.9%	22.3%	19.2%	30.8%	23.6%	20.4%	30.9%	17.6%	14.1%	28.3%	23.8%	20.3%	32.3%
AG 85–89	27.7%	22.2%	36.4%	23.7%	20.0%	33.5%	35.6%	30.4%	48.9%	29.2%	25.0%	42.3%	30.7%	29.5%	33.7%	25.7%	22.1%	38.5%	30.7%	26.7%	41.5%
AG 90–94	35.6%	27.2%	48.8%	31.8%	28.2%	42.4%	43.5%	38.2%	57.5%	37.7%	34.6%	51.7%	38.9%	36.1%	46.2%	35.2%	31.2%	48.4%	35.4%	31.8%	46.1%
AG 95+	46.6%	42.9%	56.1%	43.7%	41.3%	53.1%	58.6%	55.6%	70.0%	49.2%	47.1%	61.5%	42.4%	40.4%	50.0%	43.5%	40.0%	63.2%	44.1%	41.7%	53.9%
*Percentage of decedents in hospital*																					
All age groups	6.7%	7.1%	6.3%	21.3%	20.4%	22.9%	36.5%	34.6%	39.9%	21.3%	19.4%	25.4%	12.0%	10.3%	15.2%	40.0%	35.2%	49.7%	20.5%	19.2%	22.9%
AG 65–69	10.0%	0.0%	16.7%	20.8%	14.7%	26.3%	‐	‐	‐	25.0%	15.0%	36.1%	‐	‐	‐	36.4%	25.0%	66.7%	27.2%	25.6%	29.2%
AG 70–74	0.0%	0.0%	0.0%	13.0%	11.6%	14.8%	‐	‐	‐	22.1%	21.7%	22.4%	‐	‐	‐	44.4%	42.9%	50.0%	27.3%	29.5%	24.2%
AG 75–79	7.5%	6.9%	8.3%	18.6%	15.8%	22.0%	‐	‐	‐	20.7%	20.3%	21.5%	9.1%	‐	‐	42.9%	25.0%	66.7%	24.2%	22.6%	26.3%
AG 80–84	6.2%	6.3%	6.1%	21.3%	18.6%	25.0%	33.9%	32.3%	36.1%	18.7%	18.5%	19.1%	17.8%	17.9%	17.6%	39.4%	44.2%	32.1%	21.2%	20.1%	22.8%
AG 85–89	5.8%	6.9%	4.7%	20.9%	21.2%	20.5%	38.4%	36.3%	41.8%	19.8%	16.2%	26.6%	8.2%	5.3%	14.3%	43.9%	35.9%	59.6%	20.7%	19.7%	22.4%
AG 90–94	6.5%	6.9%	6.1%	23.0%	22.4%	24.2%	42.3%	39.8%	46.6%	22.6%	20.6%	28.7%	10.6%	10.4%	12.5%	37.2%	31.3%	50.0%	18.1%	16.3%	22.0%
AG 95+	11.8%	11.1%	13.0%	24.4%	23.3%	27.5%	40.2%	38.1%	46.4%	25.6%	23.4%	36.1%	14.3%	19.0%	‐	35.2%	38.1%	25.0%	15.2%	14.6%	17.2%

*Note*: AG = age group; F = female; M = male; "‐" = not reported due to data protection reasons.

Abbreviation: ICCONIC, International Collaborative on Costs, Outcomes, and Needs in Care.

The frail older adult with hip fracture was characterized by high multimorbidity and high utilization of services across all countries. Within the last 180 days before death, they received on average across countries 11.4 different drug substances (range: 7.3 [AU] to 15.8 [NZ]). On average, they had 1.81 hospitalizations (range: 1.56 [AU] to 2.20 [EN]) and spent 21.5 days in hospital (range: 13.1 [NZ] to 32.5 [EN]). During the same period, the decedents visited specialists, on average, 3.5 times (range: 0.95 [NZ] to 6.4 [ES]), primary care doctors, 7.3 times (range: 2.2 [US] to 11.7 [ES]), and emergency departments, 0.75 times (range: 0.15 [NZ] to 2.24 [ES]; see Table 2; Appendices 3 and 4 show numbers by sex).

**TABLE 2:** Utilization and spending by country during 30, 90, and 180 days before death

	Utilization						Spending [in US$]				
	Acute hospital admissions	Days in hospital	Emergency department visits	Specialist visits	Primary care visits	Drug prescriptions	Hospital spending	Emergency department spending	Specialist spending	Primary care spending	Drug spending
30 days before death											
Australia	0.45	4.2	0.07	0.78	2.1	2.0	6937	39	112	107	120
Canada	0.84	9.2	0.16	0.78	2.5	6.6	11,611	60	94	187	219
England	0.94	12.7	0.08	0.40	2.1	6.0	7982	48	83	87	212
Germany	0.93	9.6	‐	0.58	0.6	3.6	8094	‐	88	102	238
New Zealand	0.68	4.9	0.03	0.13	‐	8.7	4942	8	21	‐	143
Spain	1.03	14.8	1.05	2.10	2.7	5.2	9089	226	284	174	236
United States	0.77	5.4	0.24	0.71	0.3	3.6	11,109	276	241	87	267
90 days before death											
Australia	0.97	8.7	0.16	1.73	5.2	4.4	13,805	79	209	247	372
Canada	1.28	17.1	0.40	2.14	5.9	10.1	19,356	135	242	389	648
England	1.54	24.2	0.18	1.40	4.7	10.0	12,909	97	259	187	660
Germany	1.51	19.5	‐	1.61	1.5	7.9	14,092	‐	249	318	688
New Zealand	1.18	9.7	0.08	0.46	‐	13.1	9485	19	88	‐	323
Spain	1.38	21.7	1.68	4.22	6.8	9.1	13,802	363	467	423	609
United States	1.31	9.7	0.52	2.29	1.0	7.5	18,867	625	977	321	914
180 days before death											
Australia	1.56	13.3	0.28	2.85	9.4	7.3	20,200	129	329	422	722
Canada	1.72	24.0	0.70	4.15	9.5	12.7	25,839	229	456	626	1291
England	2.20	32.5	0.31	2.65	7.9	12.2	16,503	158	504	312	1325
Germany	2.07	27.5	‐	3.13	2.8	10.8	18,931	‐	492	594	1274
New Zealand	1.64	13.1	0.15	0.95	‐	15.8	12,827	33	190	‐	576
Spain	1.71	26.6	2.24	6.40	11.7	10.9	17,427	483	689	712	1193
United States	1.80	13.2	0.82	4.66	2.2	10.4	25,336	997	2156	696	1923

Across all countries, we observed relatively higher expenses in the time interval closer to the date of death. Canada had the highest hospital costs within 180 days (mean of US$ 25,839) with 44.9% of this amount incurred in the last 30 days before death. All other countries similarly incurred the highest costs related to acute hospital care within the last 30 days before death, ranging from 34.3% in Australia to 52.2% in Spain. For the other spending categories, concentration of costs in the last 30 days before death was much less pronounced.

### 4.2 Regression results

Regression results with country‐specific baseline utilization and spending, effects of sex, age gradients, and time effects are presented in Table 3. Root mean squared error ranges between 0.20 for emergency department visits and 33,120 for hospital spending. Adjusted *R*‐squared ranges between 0.66 for specialist visits and 0.98 for drug prescriptions. The estimates for baseline utilization and spending were mainly positive and, for most countries, across all estimations, significantly different from zero. This suggests that utilization and spending not only depend on the number of days, but that there is a nonlinear relationship between days before death and utilization and spending. In some cases, females seem to receive less care than men. Females had significantly less hospitalizations in New Zealand (−0.25) and spent less days in hospital in Canada (−3.11) and Spain (−8.87). The sex effect on hospital expenditures is statistically significantly negative in Canada (US$ −3618), Germany (US$ −2963), and the United States (US$ −4030), while the other countries also show a negative but not significant coefficient at the 5% level. This suggests that although women have an equally high utilization, they tend to receive less expensive treatments in those countries. In the outpatient sector, the situation is ambiguous: in the United States, specialist spending is significantly lower for women (US$ −393.4), while primary care spending is higher for women in Canada (US$ 96.2) and Spain (US$ 90.8). The age gradient is almost always significantly negative for both utilization and spending, suggesting significantly lower utilization before death among older than younger people. The time effect is consistently significantly positive as utilization and spending increase with a longer the time horizon (see Table 3).

**TABLE 3:** Regression results by utilization and spending categories

Utilization	Acute hospital admissions (SE)	Days in hospital (SE)	Emergency department visits (SE)	Specialist visits (SE)	Primary care visits (SE)	Drug prescriptions (SE)
Baseline utilization						
Australia	0.41 (0.24) .	6.05 (3.73)	0.04 (0.16)	2.79 (1.7)	0.02 (1.96)	1.98 (1.07) .
Canada	1.17 (0.15)***	16.3 (2.27)***	0.22 (0.09)*	1.37 (1.03)	1.65 (0.62)**	5.6 (0.65)***
England	1.27 (0.15)***	8.99 (2.27)***	0.1 (0.09)	1.79 (1.03) .	1.05 (0.62) .	6.53 (0.65)***
Germany	1.32 (0.15)***	16.43 (2.27)***	‐	0.29 (1.03)	0.05 (0.62)	2.8 (0.65)***
New Zealand	0.6 (0.15)***	5.45 (2.27)*	0 (0.09)	0.28 (1.03)	‐	7.72 (0.65)***
Spain	0.89 (0.15)***	22.98 (2.27)***	1.14 (0.09)***	3.2 (1.03)**	1.12 (0.62) .	3.93 (0.65)***
United States	1.17 (0.24)***	11.27 (3.73)**	0.4 (0.16)*	2.1 (1.7)	0.18 (1.01)	4.47 (1.07)***
Sex effect (female = 1)						
Australia	−0.09 (0.17)	−1.45 (2.64)	−0.07 (0.111)	2.18 (1.202) .	−0.77 (0.719)	−0.57 (0.756)
Canada	−0.06 (0.1)	−3.11 (1.55)*	0.07 (0.065)	−0.23 (0.703)	0.6 (0.42)	0.82 (0.442) .
England	0.12 (0.1)	−0.46 (1.55)	0 (0.065)	−0.83 (0.703)	0.66 (0.42)	1.11 (0.442)*
Germany	−0.09 (0.1)	−2.52 (1.55)	‐	−0.36 (0.703)	−0.01 (0.42)	0.35 (0.442)
New Zealand	−0.25 (0.1)*	−2.27 (1.55)	−0.03 (0.065)	−0.21 (0.703)	‐	−0.56 (0.442)
Spain	0.01 (0.1)	−8.87 (1.55)***	0.07 (0.065)	0.78 (0.703)	1.28 (0.42)**	1.17 (0.442)**
United States	−0.15 (0.17)	−2.03 (2.64)	−0.05 (0.111)	−0.41 (1.202)	−0.02 (0.719)	0.07 (0.756)
Age effect						
Australia	−0.04 (0.02) .	−0.38 (0.39)	0.01 (0.02)	−1.122 (0.18)***	0.017 (0.02)	−0.11 (0.11)
Canada	−0.1 (0.02)***	−1.71 (0.39)***	−0.05 (0.02)**	−0.294 (0.18) .	−0.185 (0.11) .	−0.13 (0.11)
England	−0.16 (0.02)***	0.18 (0.39)	−0.015 (0.02)	−0.358 (0.18)*	−0.065 (0.11)	−0.45 (0.11)***
Germany	−0.11 (0.02)***	−1.76 (0.39)***	‐	−0.019 (0.18)	0.024 (0.11)	−0.1 (0.11)
New Zealand	0.01 (0.02)	−0.1 (0.39)	0 (0.02)	−0.034 (0.18)	‐	0.07 (0.11)
Spain	0.01 (0.02)	−1.46 (0.39)***	−0.071 (0.02)***	−0.534 (0.18)**	−0.145 (0.11)	0.06 (0.11)
United States	−0.09 (0.02)***	−1.03 (0.39)**	−0.048 (0.02)**	−0.439 (0.18)*	−0.059 (0.11)	−0.44 (0.11)***
Time effect						
Australia	0.009 (0.001)***	0.06 (0.02)**	0.001 (0.001)	0.041 (0.01)***	0.044 (0.006)***	0.034 (0.006)***
Canada	0.006 (0.001)***	0.11 (0.01)***	0.004 (0.001)***	0.025 (0.006)***	0.05 (0.003)***	0.042 (0.004)***
England	0.011 (0.001)***	0.13 (0.01)***	0.002 (0.001)**	0.02 (0.006)***	0.037 (0.003)***	0.043 (0.004)***
Germany	0.008 (0.001)***	0.13 (0.01)***	‐	0.018 (0.006)**	0.015 (0.003)***	0.048 (0.004)***
New Zealand	0.007 (0.001)***	0.06 (0.01)***	0.001 (0.001) .	0.006 (0.006)	‐	0.047 (0.004)***
Spain	0.004 (0.001)***	0.09 (0.01)***	0.007 (0.001)***	0.027 (0.006)***	0.054 (0.003)***	0.03 (0.004)***
United States	0.006 (0.001)***	0.04 (0.02)*	0.004 (0.001)***	0.029 (0.01)**	0.013 (0.006)*	0.047 (0.006)***
Root mean squared error	0.30	4.76	0.20	2.17	1.31	1.36
Adjusted *R*‐squared	0.96	0.93	0.92	0.67	0.93	0.98

Spending in US$ PPP	Hospital spending (SE)	Emergency department spending (SE)	Specialist spending (SE)	Primary care spending (SE)	Drug spending (SE)
Baseline utilization					
Australia	12,681 (2593.5)***	31.9 (46.4)	198.5 (279.7)	117.9 (76.4)	496.9 (263.5) .
Canada	21,305 (1578.3)***	73.6 (28.2)**	152.8 (170.2)	102.2 (46.5)*	403.1 (160.4)*
England	8062 (1578.3)***	56.7 (28.2)*	288.6 (170.2) .	99.4 (46.5)*	93.2 (160.4)
Germany	17,322 (1578.3)***	‐	265.3 (170.2)	−67.8 (46.5)	619.1 (160.4)***
New Zealand	5350 (1578.3)***	16 (28.2)	82 (170.2)	‐	143.6 (160.4)
Spain	3837 (1578.3)*	244.7 (28.2)***	374.6 (170.2)*	50.6 (46.5)	215.3 (160.4)
United States	22,508 (2593.5)***	519.9 (46.4)***	1977.4 (279.7)***	226.1 (76.4)**	1130.6 (263.5)***
Sex effect (female = 1)					
Australia	−2811 (1836.9)	−25.6 (32.8)	213.2 (198.1)	−45.4 (54.1)	2.1 (186.7)
Canada	−3618 (1074.4)***	16.9 (19.2)	−1.6 (115.9)	96.2 (31.6)**	−26.5 (109.2)
England	−1223 (1074.4)	0.6 (19.2)	−55.2 (115.9)	25.4 (31.6)	92.3 (109.2)
Germany	−2963 (1074.4)**	‐	−56.5 (115.9)	24.2 (31.6)	70.8 (109.2)
New Zealand	−2044 (1074.4) .	−10.2 (19.2)	−49.2 (115.9)	‐	−65.1 (109.2)
Spain	−771 (1074.4)	15 (19.2)	75.2 (115.9)	90.8 (31.6)**	131.6 (109.2)
United States	−4030 (1836.9)*	−60.8 (32.8) .	−393.4 (198.1)*	20.1 (54.1)	−156.1 (186.7)
Age effect					
Australia	−1152.6 (268.6)***	1.8 (4.8)	−87.4 (29)**	−4.9 (7.9)	−137.2 (27.3)***
Canada	−2214 (268.6)***	−14.2 (4.8)**	−33.5 (29)	−11.3 (7.9)	−105.9 (27.3)***
England	−215.6 (268.6)	−7.2 (4.8)	−66.2 (29)*	−14.8 (7.9) .	−35.3 (27.3)
Germany	−1981.8 (268.6)***	‐	−58.9 (29)*	13.7 (7.9) .	−160 (27.3)***
New Zealand	−57.9 (268.6)	−0.8 (4.8)	−14 (29)	‐	−15.4 (27.3)
Spain	1184 (268.6)***	−15.2 (4.8)**	−49.5 (29) .	−2.9 (7.9)	−55.3 (27.3)*
United States	−1925.1 (268.6)***	−68.2 (4.8)***	−434.6 (29)***	−77.9 (7.9)***	−266.3 (27.3)***
Time effect					
Australia	80.3 (14.9)***	0.5 (0.3) .	4.1 (1.6)*	1.9 (0.4)***	6.2 (1.5)***
Canada	105.7 (8.7)***	1.3 (0.2)***	2.8 (0.9)**	3.1 (0.3)***	8.4 (0.9)***
England	61.6 (8.7)***	0.8 (0.2)***	3.7 (0.9)***	1.5 (0.3)***	7.4 (0.9)***
Germany	81.3 (8.7)***	‐	3.3 (0.9)***	3.2 (0.3)***	8.9 (0.9)***
New Zealand	52.9 (8.7)***	0.1 (0.2)	1 (0.9)	‐	3.1 (0.9)***
Spain	48.7 (8.7)***	1.6 (0.2)***	2.6 (0.9)**	3.2 (0.3)***	5.7 (0.9)***
United States	77.2 (14.9)***	4.8 (0.3)***	15.2 (1.6)***	5.1 (0.4)***	12.7 (1.5)***
Root mean squared error	3311.5	59.7	357.1	98.2	336.5
Adjusted *R*‐squared	0.96	0.97	0.84	0.93	0.88

*Note*: “‐” utilization/spending category not available in this country; significance codes: “***” = 0.001, “**” = 0.01, “*” = 0.05, “.” = 0.1.

Abbreviation: SE, standard errors.

## 5 DISCUSSION

International comparisons of end‐of‐life care for frail older adults provide important insights as to how health systems manage complex populations at the end of life. Using patient‐level data linked across inpatient and outpatient health care sectors, we found important differences in how older adults with hip fracture are managed across a group of seven high‐income countries. We also shed insights into how treatment intensity differences according to age, sex, and time may explain some of the variation across countries. By comparing expenditures relative to proximity of death, policy makers may be able to identify opportunities to better allocate scarce health care resources.

A key finding of this work is the large difference across countries in the site of death, specifically whether it occurred in a hospital setting or not. Site of death is increasingly becoming an important measure of health system performance, especially as prior studies have shown that patients prefer dying at home,^46^, ^47^ something that is not the reality in most countries.^48^ In our study, England (36.5%) and Spain (40%) are among the countries with the most decedents dying in hospital. Australia and New Zealand, on the other hand, observed the lowest rate of in hospital‐based deaths at 6.7% and 12%. Similar patterns regarding site of care have also been found elsewhere.^7^, ^49^ The high rate in England seems to be related to unplanned hospital care at the end of life,^50^ while the low rate in New Zealand may be attributed to a historically strong focus on hospice and end‐of‐life care, which limits the number of days a patient may spend in a hospital setting in their final days of life.^51^ Indeed, end‐of‐life care in New Zealand tends to be provided in the home with the support of homecare, medical, and hospice services. In cases where a higher level of care may be needed, patients are cared for in a hospice facility or an elderly care facility designed to provide specialized end‐of‐life support services.^52^ Furthermore, it is also important to not only consider the site of death but also where the majority of end‐of‐life care took place prior to the death.^53^ According to our data, the number of days spent in hospital within the last month is strongly correlated with the rate of decedents dying in the hospital, that is, the more days spent in hospital care, the higher the in‐hospital mortality rate. Further support for specialized and community palliative care services may offer patients and their carers more choice in deciding the most appropriate site of death for them.^47^

We also observed important differences in the treatment by health care sector at the end of life, which is a novel contribution to previous international comparisons on end‐of‐life care. It was interesting to observe that primary care visits play a much smaller role in Germany (2.8) and the United States (2.2) than in the other countries, where the average number of visits across Australia, Canada, England, and Spain was 9.6 visits (range 9.4–11.7). With regard to specialist visits, the United States is second with 4.7 visits, while in Germany, an average of 3.1 specialist visits take place within the last 180 days of life. The low number in Germany can be attributed to the reimbursement system, where many services in the outpatient sector are flat rate and bundled over a quarter. In the United States, however, it seems that care at the end of life occurs more with specialists than with primary care, which is a fundamental contrast to other countries (e.g., Australia, Canada, England, and Spain) where the primary care physician appears to assume a more prominent role. This is consistent with overall patterns of utilization.^54^ Prescription drug use was high across all countries, ranging from 7.3 unique type of scripts in Australia to 15.8 in New Zealand. Spending on drugs, however, was relatively modest and ranged from US$ 576 in New Zealand to US$ 1923 in the United States in last 180 days of life.

Hospice use is organized very differently across countries and difficult to capture with our administrative data that are underlined by the divergent data that are provided by Germany and the United States (see Appendix 5 for utilization and spending). According to our data, end‐of‐life patients spend, on average, 0.16 days in hospice in Germany, while they spend 8.3 days in hospice in the United States. In terms of hospital days, the situation is the opposite: in Germany, decedents spend 9.6 days, and in the United States, only 5.4 days in hospital during their last phase of life. In Australia, England, Germany, and Spain, the decedents are very much supported by palliative care teams that are based in the reference hospitals or are part of the primary care organization and therefore included in the inpatient and specialist costs.^55^ Similarly, in New Zealand, end‐of‐life care is provided in the home, with the support of homecare, medical, and hospice services, in a hospice if there are comorbidities that require active care, or in an aged care facility, which is designed to provide specialized end‐of‐life support services. However, the cost of aged care accommodation is often serviced by the patient, subject to an asset assessment, with only limited costs coming from the public sector. These different organizational and reimbursement arrangements make a direct comparison difficult, and our study based on administrative data should be complemented by qualitative research to explore the variation in the organization of hospice care further.

In most international comparisons, the United States typically has the highest health care costs.^56^, ^57^ Interestingly, this seems not to be the case for end‐of‐life care in the acute hospital setting, while the United States still has the highest health care costs across all other spending categories analyzed in this study. This may also be reflective of the fact that the use of advance care planning, and of end‐of‐life directives, may be more limited in the United States as compared to other countries. In previous work, Gupta et al.^58^ reported that among US Medicare beneficiaries, the presence of an advanced care plan was associated with less intensive health care utilization around the end of life. Similar to our current findings reported, Bekelman et al.^7^ found in their study of cancer patients that Canada had the highest end‐of‐life hospital costs, while Germany had slightly lower costs than Canada. This is not because the United States has fundamentally lower hospital costs but may reflect fewer inpatient hospital days in the United States prior to death and likely more time during end‐of‐life care spent in postacute rehabilitative facilities.^54^

We also encountered important differences across some countries in the treatment of people by age. Canada, Germany, and the United States had the highest negative age gradients, that is, on average, these countries spend around US$ 2000 less per older age group. In contrast, the age effect on spending did not play a role in New Zealand and England across all health care services. This negative age gradient indicates that countries discriminate against age—whether this addresses the patient needs to or due to efficiency considerations. It is likely that spending is managed by differentiation of the place of service delivery during the trajectories of care. The study by Wodchis et al.^59^ on trajectories of care suggests that patients are primarily transferred to home settings, that is, home, home rehab, home nursing, and so forth, prior to death. Inpatient rehab plays a subordinate role for decedents because if there is no chance of recovery, they are not moved to these facilities; in Australia, Canada, and Germany, for example, a decedent spends on average a very short time in an inpatient rehabilitation unit during the last 30 days before death (AU 0.86, CA 1.06, and DE 0.37 days). In the United States, however, a decedent spends on average 7.41 days in inpatient rehabilitation, especially in skilled nursing homes. This effect, however, converges when viewed over 90 and 180 days (see Appendix 5 for utilization and spending). There are several reasons why this might be the case. Patients have different accessibility to postacute rehabilitative care and/or long‐term care, which may influence the amount of time spent in the hospital setting by age. In England, the National Health Service does not cover these services, which limits the ability of hospitals to discharge patients needing this type of care. In the United States, patients are likely to be quickly discharged to postacute rehabilitative care, while in Canada, patients have access to long‐term care. New Zealand, on the other hand, had relatively consistent spending through the age groups, which as noted above, may be due to the country's concerted and coordinated application of elder care assessment services and support.

In part, the variation in use and costs across countries in each health care sector (e.g., hospitals) may be driven by differences in the roles and functions of that sector. For example, rehabilitation in some countries may be a part of a hospital's routine functions—whereas in other countries, much of this care may be provided in specialized clinics or in the community. This implies that rehabilitation costs will be incurred in the hospital sector for some countries, but in other countries, it may be registered as an outpatient care. Such differences in the roles and functions may be important drivers of the variation seen between countries. The results reported in this article provide an accurate description of costs and use within each sector, but further work is needed to examine the reasons for that variation—which includes further exploration of the roles and functions of each sector as well as full data capture.

Finally, we observed that some countries spend less on treating women before death. Specifically, while men and women generally receive similar care in the outpatient setting, there appears to be some differences in the type of care provided in the acute care setting, with males receiving more expensive acute hospital end‐of‐life care across countries. There are some potential factors that might explain this finding. First, prior work has suggested that women may have a lower biological age than men and may therefore be less frail.^60^ There is also some evidence that suggests men enter the last phase of life sicker than women. For example, in Germany, male decedents scored a higher Elixhauser mortality score^61^ than women. Second, it is possible that there may be differences in preference of high‐intensity treatments by gender or in the offering of such treatments by clinicians. This effect has been shown for other conditions, for example, Sharma et al.^29^ find that men with advanced cancers are more likely than women to receive aggressive, nonbeneficial care in the intensive care unit near death. Due to the paucity of international studies on this topic, there is a limited capacity to compare our results to others. In the few studies available, there is some agreement in the results. In comparison to cancer patients, for example, there were similar costs associated with acute care costs but, perhaps not surprisingly, a much higher costs on outpatient care compared to the patients in our sample.^7^ Further, in our study, we find that the United States spends substantially more for patients at the end of life after a hip fracture, which is consistent with the fact that they spend more overall for this population and other populations like people hospitalized with heart failure, even after adjusting for similar levels of utilization. Our findings confirm that the United States has high prices per unit across multiple care domains.

### 5.1 Limitations

The study has limitations. First, we had to rely on a federated analysis approach due to data protection regulations. Across countries, there are differences in how data are structured and collected, which may yield differences in the variables examined in this study. However, we used a patient vignette with specific diagnostic codes that are commonly used that should limit potential biases and deviations across countries. Second, the federated approach did not allow us to perform the estimations with individual‐level data, which lower the efficiency of our model; however, the coefficients should remain unbiased. The small number of data points may also lead to a technical overfitting of the regression. However, given that the data points are based on thousands of observations, it still allows for a generalizability of the conclusions. Third, the method applied ignores that spending may be frontloaded during hospital stays in case of accruals (as illustrated in Figure 1B). This leads to an overestimation of the actual hospital costs incurred, most notably in the last 30 days before death. This is a problem of almost all claims data analyses and fades the longer the observation period is. Fourth, we used only a limited number of utilization and spending categories, focusing on the most important categories available and comparable across countries. Further research should invest additional effort in detailing and identifying further categories and making them comparable to complement our picture of the provision of end‐of‐life care. Fifth, when comparing absolute spending across countries, we have to acknowledge that we compare prices but do not account for the different economics of care delivery. We explored the economic implications of different patterns of resource use in the study of Lorenzoni et al.^62^ Lastly, we were unable to determine whether the differences in spending and utilization within and between countries are due to differences in responsiveness to the populations' demands, better allocative efficiency by countries, or whether the end‐of‐life spending was potentially wasteful.^25^ Further, we must be very careful with any causal conclusions based on the data. Without taking the endogeneity issue and the health system structures into account, it is very difficult to interpret the effects.^37^ Despite our significant progress in making the results as comparable as possible, great care still needs to be taken in drawing conclusions on the reasons for the cross‐country variations.

## 6 CONCLUSION

Across a group of seven high‐income countries, we found important variations in end‐of‐life care for patients who sustained a hip fracture, with some differences explained by sex and age. Our work sheds important insights that may help ongoing health policy discussions on equity, efficiency, and reimbursement in health care systems. However, while our analysis has limitations, improved data capture and availability might substantially increase the explanatory value of international comparisons in the future.

## DISCLAIMER

The opinions, results, and conclusions reported in this paper are those of the authors and are independent from funding sources. The funders had no role in study design, data collection and analysis, decision to publish, or preparation of the manuscript.

The 45 and Up survey data used to represent Australia oversample people older than 80 years and residents of rural and remote areas (45 and Up Study Collaborators; 2008). The 45 and Up Study had a response rate of 18%, so the cohort might not be representative of the NSW population. Also, the survey focuses on NSW and may not be representative of the national sample for the same age group.

The results from New Zealand are not official statistics. They have been created for research purposes from the Integrated Data Infrastructure (IDI), which is carefully managed by Stats NZ. For more information about the IDI, please visit www.stats.govt.nz/integrated-data.

## Supporting information


**Appendix S1** Supporting information
