# Supplementary material for: International comparison of spending and utilization at the end of life for hip fracture patients
Source: Health Serv Res. 2021 Sep 7;56(Suppl 3):1370–82. doi: 10.1111/1475-6773.13734 (PMC8579204; doi:10.1111/1475-6773.13734)
Supplement: Supplementary file 1 — Appendix S1 Supporting information [file HESR-56-1370-s001.docx]

# Appendix

Appendix 1: Country Dataset Information

The ICCONIC collaborative uses linked 2015-17 patient-level data from multiple care settings - spanning primary care, specialty services, acute hospital care, and post-acute care - from 11 countries: Australia, Canada, England, France, Germany, Netherlands, New Zealand, Spain, Sweden, Switzerland, and the United States. The data includes information on patient characteristics, comorbidities, utilization, spending and outcomes for two patient personas: a frail elder, identified as an individual aged 65+ years with a hospital admission for hip fracture (the hip fracture persona); and a complex, multi-morbid patient, identified as an individual aged 65-90 admitted to hospital with a diagnosis of congestive heart failure and type 2 diabetes as a comorbidity (the congestive heart failure and diabetes persona). For a detailed overview of the methodology employed to collect the data, see the methodological study^28^.

| **Country** | **Datasets** |
| --- | --- |
| Australia | - Sax Institute’s 45 and Up study |
| Canada | - Administrative claims data of the province of Ontario from the Ontario Ministry of Health and the Canadian Institute for Health Information through the Institute for Clinical Evaluative Sciences (ICES) |
| England | - Primary care data from the Clinical Practice Research Datalink (CPRD)  linked to secondary care data from Hospital Episode Statistics (HES) and Office for National Statistics (ONS) death register |
| Germany | - Administrative data of a large, nationally active health insurance with more than 8m enrollees (BARMER) (includes utilization/costs of all sectors that are paid by health insurance) |
| New Zealand | - The Integrated Data Infrastructure (IDI) - The National Minimum Dataset (NMDS) (hospital admissions data) - The pharmaceutical collection (medication dispensing data) - The National Non-Admitted Patient Collection  (NNPAC) (outpatient data) |
| Spain | - Base de datos de usuario (National Health Service users dataset including insurees admin data) - OMI-AP (primary care electronic health records) - Conjunto Mínimo Básico de Datos (CMBD) (admin data  for hospital discharges and outpatient contacts) - Sistema de Información Hospitalaria  (outpatient visits to specialized care) - Receta Electrónica (e-Prescription files) - Facturación Recetas (billing files of over-the-counter prescriptions) - Puesto Clínico Hospitalario de Urgencias (emergency care contacts) |
| United States | - Medicare fee-for-service (FFS) data, 20% sample of all patients age 65 years or older |

Appendix 2: Diagnostic Codes Used to Identify Hip Fracture Patients

| **NAM Priority Population** | **Identified High-Need Patient Personas for Comparison** | **Age Group** | **Identification with Diagnostic Codes*** |
| --- | --- | --- | --- |
| **Frail older person** | Older person with hip fracture | 65 years and older | Diagnoses  *-S72.0: Fracture of neck of femur*  *-S72.1: Pertrochanteric fracture*  *-S72.2: Subtrochanteric fracture*  Procedures (using country-specific codes):  *-Total hip replacement*  *-Partial hip replacement*  *-Osteosynthesis/pinning* |

***** Across most countries, the diagnostic classification system of ICD-10-WHO codes were used. Spain used ICD-9 codes while the Netherlands used a customized approach to identify relevant codes using input from clinical experts in private insurer data. Australia used Australian Classification of Health Interventions (ACHI) codes to capture the procedures.

Appendix 3: Utilization and spending by country during 30, 90, and 180 days before death: females

|  |  | **Utilization** | |  |  |  |  |  | **Spending [in US$]** | |  |  |  |
| --- | --- | --- | --- | --- | --- | --- | --- | --- | --- | --- | --- | --- | --- |
|  |  | **Acute hospital admissions** | **Days in  hospital** | **Emergency department visits** | **Specialist visits** | **Primary care visits** | **Drug prescriptions** |  | **Hospital Spending** | **Emergency department spending** | **Specialist spending** | **Primary care spending** | **Drug spending** |
| **30 days before death** | | |  |  |  |  |  |  |  |  |  |  |  |
|  | Australia | 0.42 | 3.8 | 0.07 | 0.86 | 2.2 | 2.0 |  | 6,240 | 44 | 117 | 115 | 112 |
|  | Canada | 0.81 | 8.6 | 0.16 | 0.76 | 2.5 | 6.8 |  | 10,732 | 59 | 91 | 195 | 222 |
|  | England | 0.93 | 12.2 | 0.07 | 0.30 | 2.2 | 6.4 |  | 7,511 | 43 | 70 | 92 | 228 |
|  | Germany | 0.87 | 8.8 | -- | 0.55 | 0.6 | 3.7 |  | 7,194 | -- | 83 | 106 | 238 |
|  | New Zealand | 0.63 | 4.4 | 0.02 | 0.13 | -- | 8.5 |  | 4,401 | 5 | 18 | -- | 141 |
|  | Spain | 1.08 | 12.6 | 1.12 | 2.70 | 3.2 | 5.3 |  | 8,699 | 241 | 348 | 205 | 247 |
|  | United States | 0.73 | 4.9 | 0.23 | 0.64 | 0.3 | 3.7 |  | 10,055 | 260 | 194 | 82 | 260 |
| **90 days before death** | | |  |  |  |  |  |  |  |  |  |  |  |
|  | Australia | 0.87 | 7.5 | 0.14 | 1.84 | 5.4 | 4.6 |  | 12,735 | 75 | 219 | 257 | 341 |
|  | Canada | 1.24 | 16.0 | 0.41 | 2.03 | 5.8 | 10.2 |  | 17,863 | 137 | 232 | 399 | 646 |
|  | England | 1.57 | 23.9 | 0.16 | 0.96 | 4.9 | 10.2 |  | 12,599 | 86 | 200 | 192 | 686 |
|  | Germany | 1.45 | 18.3 | -- | 1.49 | 1.6 | 8.0 |  | 12,966 | -- | 225 | 331 | 683 |
|  | New Zealand | 1.11 | 8.9 | 0.07 | 0.42 | -- | 13.0 |  | 8,717 | 15 | 74 | -- | 312 |
|  | Spain | 1.38 | 18.6 | 1.67 | 4.36 | 6.8 | 8.8 |  | 12,973 | 360 | 476 | 425 | 608 |
|  | United States | 1.25 | 8.8 | 0.49 | 2.04 | 1.0 | 7.6 |  | 17,340 | 588 | 765 | 310 | 856 |
| **180 days before death** | | |  |  |  |  |  |  |  |  |  |  |  |
|  | Australia | 1.49 | 11.7 | 0.27 | 2.80 | 9.7 | 7.6 |  | 19,008 | 126 | 332 | 442 | 673 |
|  | Canada | 1.68 | 22.6 | 0.71 | 3.89 | 9.3 | 12.9 |  | 24,176 | 232 | 428 | 637 | 1,252 |
|  | England | 2.23 | 32.2 | 0.29 | 1.99 | 8.2 | 12.5 |  | 16,069 | 146 | 409 | 318 | 1,361 |
|  | Germany | 2.01 | 26.4 | -- | 2.88 | 2.8 | 10.8 |  | 17,828 | -- | 411 | 609 | 1,216 |
|  | New Zealand | 1.55 | 12.3 | 0.14 | 0.89 | -- | 15.7 |  | 12,145 | 30 | 173 | -- | 534 |
|  | Spain | 1.68 | 21.4 | 2.16 | 6.58 | 11.6 | 10.9 |  | 16,339 | 467 | 706 | 713 | 1,229 |
|  | United States | 1.73 | 12.2 | 0.78 | 4.18 | 2.1 | 10.4 |  | 23,488 | 954 | 1,766 | 671 | 1,765 |

Appendix 4: Utilization and spending by country during 30, 90, and 180 days before death: males

|  |  | **Utilization** | |  |  |  |  |  | **Spending [in US$]** | |  |  |  |
| --- | --- | --- | --- | --- | --- | --- | --- | --- | --- | --- | --- | --- | --- |
|  |  | **Acute hospital admissions** | **Days in  hospital** | **Emergency department visits** | **Specialist visits** | **Primary care visits** | **Drug prescriptions** |  | **Hospital  Spending** | **Emergency department spending** | **Specialist spending** | **Primary care spending** | **Drug  spending** |
| **30 days before death** | | |  |  |  |  |  |  |  |  |  |  |  |
|  | Australia | 0.49 | 4.6 | 0.06 | 0.70 | 1.9 | 2.0 |  | 7,652 | 34 | 108 | 99 | 129 |
|  | Canada | 0.90 | 10.1 | 0.16 | 0.80 | 2.5 | 6.3 |  | 13,069 | 60 | 99 | 175 | 213 |
|  | England | 0.95 | 13.6 | 0.10 | 0.58 | 1.9 | 5.4 |  | 8,792 | 56 | 105 | 79 | 186 |
|  | Germany | 1.06 | 11.5 | -- | 0.64 | 0.6 | 3.3 |  | 10,043 | -- | 99 | 95 | 238 |
|  | New Zealand | 0.77 | 5.8 | 0.05 | 0.14 | -- | 8.9 |  | 5,965 | 13 | 28 | -- | 147 |
|  | Spain | 0.97 | 18.8 | 0.98 | 1.51 | 2.2 | 5.0 |  | 9,478 | 211 | 221 | 142 | 224 |
|  | United States | 0.84 | 6.3 | 0.26 | 0.83 | 0.3 | 3.5 |  | 12,965 | 306 | 324 | 95 | 282 |
| **90 days before death** | | |  |  |  |  |  |  |  |  |  |  |  |
|  | Australia | 1.06 | 10.0 | 0.18 | 1.61 | 5.0 | 4.3 |  | 14,901 | 83 | 199 | 237 | 404 |
|  | Canada | 1.36 | 19.1 | 0.40 | 2.31 | 5.9 | 9.8 |  | 21,828 | 131 | 259 | 371 | 650 |
|  | England | 1.50 | 24.8 | 0.22 | 2.17 | 4.3 | 9.6 |  | 13,442 | 115 | 361 | 179 | 617 |
|  | Germany | 1.64 | 21.9 | -- | 1.85 | 1.5 | 7.7 |  | 16,529 | -- | 301 | 291 | 699 |
|  | New Zealand | 1.33 | 11.2 | 0.11 | 0.54 | -- | 13.2 |  | 10,937 | 27 | 115 | -- | 342 |
|  | Spain | 1.44 | 31.8 | 1.61 | 3.95 | 5.5 | 7.8 |  | 14,031 | 346 | 442 | 336 | 520 |
|  | United States | 1.41 | 11.1 | 0.57 | 2.73 | 1.1 | 7.4 |  | 21,552 | 688 | 1,349 | 339 | 1,034 |
| **180 days before death** | | |  |  |  |  |  |  |  |  |  |  |  |
|  | Australia | 1.63 | 14.9 | 0.29 | 2.91 | 9.1 | 6.9 |  | 21,423 | 133 | 327 | 402 | 773 |
|  | Canada | 1.81 | 26.5 | 0.69 | 4.57 | 9.9 | 12.4 |  | 28,593 | 222 | 503 | 606 | 1,355 |
|  | England | 2.14 | 33.1 | 0.35 | 3.79 | 7.5 | 11.7 |  | 17,250 | 178 | 667 | 302 | 1,264 |
|  | Germany | 2.21 | 30.0 | -- | 3.69 | 2.8 | 10.7 |  | 21,316 | -- | 667 | 561 | 1,400 |
|  | New Zealand | 1.82 | 14.8 | 0.16 | 1.07 | -- | 15.9 |  | 14,116 | 38 | 224 | -- | 656 |
|  | Spain | 1.71 | 48.1 | 2.16 | 5.83 | 10.0 | 8.8 |  | 16,817 | 466 | 642 | 592 | 946 |
|  | United States | 1.92 | 15.1 | 0.89 | 5.49 | 2.3 | 10.3 |  | 28,587 | 1,073 | 2,843 | 741 | 2,251 |

Appendix 5: Utilization and spending by country during 30, 90, and 180 days before death: inpatient post-acute rehabilitative and hospice care

|  |  |  | **Utilization** |  |  | **Spending [in US$]** |  |
| --- | --- | --- | --- | --- | --- | --- | --- |
|  |  |  | **Days in inpatient post-acute rehabilitative care** | **Days in hospice care** |  | **Inpatient post-acute rehabilitative care** | **Hospice care** |
| **30 days before death** | |  |  |  |  |  |  |
|  | Australia |  | 0.86 | - |  | - | - |
|  | Canada |  | 1.06 | - |  | 1,782 | - |
|  | Germany |  | 0.37 | 0.16 |  | 83 | 36 |
|  | United States |  | 7.41 | 8.30 |  | 3,853 | 1,868 |
| **90 days before death** | |  |  |  |  |  |  |
|  | Australia |  | 3.02 | - |  | - | - |
|  | Canada |  | 5.30 | - |  | 4,310 | - |
|  | Germany |  | 1.42 | 0.25 |  | 318 | 60 |
|  | United States |  | 20.06 | 15.03 |  | 10,405 | 3,052 |
| **180 days before death** | |  |  |  |  |  |  |
|  | Australia |  | 6.06 | - |  | - | - |
|  | Canada |  | 10.64 | - |  | 6,812 | - |
|  | Germany |  | 2.59 | 0.34 |  | 583 | 80 |
|  | United States |  | 33.19 | 20.43 |  | 17,154 | 3,971 |
